# Pathology and Treatment of Anæmia

**Published:** 1858

**Authors:** H. P. Ayres

**Affiliations:** Fort Wayne, Indiana


*Pathology aud Treatment of Anœmia.* By H. P. Ayres, M. D., of Fort Wayne, Indiana.

While medical men in this country have been lavish in contributing facts respecting the pathology and treatment of many diseases, anæmia has been neglected. But rarely do we find in medical journals a paper respecting this interesting, and often tedious affliction; interesting, not only as to the varied phenomena presented, but, also, from the fact that it generally attacks the fairest portion of God's creation.

It may appear presumptive to offer theory or give opinions on subjects which have engrossed the attention, and called forth the untiring investigation of great minds since the days of Hippocrates. But, however we may differ in our views from the multiplicity of opinions which have been advanced, we will constitute but another bark on the wide sea of theory and research.

Anæmia! what is it, and how shall we treat it?

Excessive evacuations, hemorrhage, poor food, impure air, malaria, tubercles, diseased uterine action, and a variety of other troubles, have been assigned as its causes.

I have known it to appear when the appetite was good, and the secretions of the system apparently healthy; where the person was well and warmly clad, and when poorly clad; where there was a scrofulous diathesis, and where there was a freedom therefrom; in hopeful dispositions, and in dispositions of a melancholic nature; in buoyancy and prosperity, in suffering and destitution: in a word, I am satisfied no particular disposition, circumstance, or situation will secure individuals against the attacks of this disease.

The revelations which the microscope has made, respecting the various conditions of the blood in disease, the gradual, and oftentimes insiduous appearance of the trouble, even when surrounding circumstances would contra-indicate the possibility of anæmia, together with its sudden manifestations, in which all the powers of life appear suddenly to decay, and the patient, from hope and happiness, is plunged into gloomy despair, induces me to think anæmia is absolutely as primitive a disease of the blood-globules as is pneumonia of the lungs, or metritis of the womb.

There are probably exciting causes to all diseases, where they are not the result of the natural tendency in the organism to decay; but these causes are oftentimes hidden and obscure, immediate or remote.

The same reasoning is true in anæmia. There may be immediate or remote causes; these causes may act upon the globules of the blood, depriving them of their proper nourishment, and consequently destroying their healthiness, which in turn injures and changes the functions of the whole economy. The experiments of Prevost have demonstrated that the red globules of the blood are alone capable of restoring vigor and health to an exsanguine subject. Gavarret has clearly shown that health and beauty are dependent upon the same causes. Liebig says, "by the blood-globules, oxygen is conveyed to the various textures of the body;" hence we conclude that in the appearance of anæmic symptoms we have manifestations of a diseased state of this portion of the blood.

The blood-globule is a unit in itself, and as such must have its own healthy circulation and nourishment; and any causes depriving it of a healthy existence, must necessarily render it unfit to supply the waste and decay of the body. I do not wish to be understood as taking the position of Dollinger, of Germany, or in the least degree embracing his theory, further than he believed in the individual organization of the globules of the blood. This independent organization implies a liability to an individual disease, and such do we suppose is the case in anæmia. It is a disease of the blood globule.

The microscope reveals a jagged, serrated, and broken state of the globules in many diseases. We may trace the matter back, and say it is from the want of a proper decarbonized state of the blood, defective secretion of the gastric juice, vitiated chyle, or any other functional derangement; and the reasoning will be no more correct than to say, the globules may be primarily diseased. By not supplying a sufficient amount of nourishment to the various organs of the body, the functions of the latter are deranged. This derangement but serves to perpetuate and increase the hitherto existing disease of the blood, which becomes more and more impoverished. Thus a chain or circle of disease is formed, the only result of which is a continued wasting of the whole economy. Where the first departure from health in the globule is, may ever be involved in the obscurity which has overshadowed the origin or beginning of the globule. To advance a theory that the germ of the globule is formed in the lacteal system, that incubation takes place in the mesentery, that the ova, thus in its forming state, is taken up and commingled with the blood of the body, as it passes through the heart into the lungs, where the oxygen from the atmosphere fully developes and prepares it to repair the wastes which have taken place in the organism, would be no more inconsistent or untenable, than to say the membrana commune is their origin or the instrument of their production. The former we believe. Now, the generative process may be imperfect, the incubation may be unhealthy, the full developement may be deficient, the health of the globule may be affected, or its life may be too short. Let the defect be where it may, the globule is involved. While I think anæmia is the opposite of hyperæmia, I do not think the disease we call anæmia is simply a poor state of the blood, but a diseased state of the globule. This diseased state may result in a marasmus and the entire decay of the globule, in which case the anæmia may be more insidious. A more aggravated trouble may occur to the blood, by poison being absorbed from a diseased spleen, suppressed catamenia, low types of fever, vitiated liquor sanguinis, or other causes, by which the anæmia may be ushered in more rapidly. Some cases of anæmia appear to be ushered in by a sudden gangrenous condition of the blood, in which the whole mass of globules appear to be broken up, and a putrescent state of the blood, rapidly supervening, and made apparent from the fœtid perspiration, black and disgusting fæces, offensive urine, bad breath, petechia, fetid eructations, and hiccough.

But how can disease be attributed to the blood-globules, in severe anæmia, when they are not found in the circulation, or, at least, where but a small part of the blood is globules? I answer, the functional derangement which the vitiated blood has produced in the stomach, lacteal system, liver, lungs, and nervous centres, has measurably suspended the generation and development of this part of the blood.

Is there a difference in the pathology of anæmia and chlorosis? Cabanis, M. Roche, Blundell, Fox, Colombat, Ramsbotham, Brown, Velpeau, Meigs, and others, differ in their views of the two affections. One attributes them to a diseased liver, others to nervous irritation, defective action of the nervous centre, derangement of the menstrual function, and a variety of other causes; but the arguments are no stronger in favor of these various causes, than that these assigned causes are but the results of a diseased blood, which may be evinced by either trouble. True, in chlorosis there is a want of the catamenia, or, if it exists, it is pale and vitiated. It is also true, that when the catamenia improves, there are symptoms of returning health; but is the returning health from a healthy menstrual flux, or is it not better pathology to say, the healthy catamenia is the result of a healthier state of the blood, which has vivified the hitherto dormant ovaries?

However we may differ in our opinions from others, we cannot but conclude, that the pathology of chlorosis and anæmia are the same, both having the same anatomical seat, and that the varied phenomena observable in depraved functional derangements, are oftener consequences than exciting causes. The microscope shows a similarity in the globules in the two affections; and I am satisfied, as observation is extended, there will be found a still closer analogy. May it not be true, that chlorosis in girls does not oftener assume all the phenomena of anæmia, from the fact that the constituent parts of the blood are materially changed in quantity and quality, after impregnation, and particularly after childbirth?

As I have remarked in regard to anæmia, may there not be in chlorosis an atrophy—a consumption of the blood-globules—which may exist not only for weeks, but for months and years, before the patient's health is materially affected? and the ultimaum of such cases has often been, a full development of all the phenomena of anæmia.

The entire nervous excitement which characterizes severe and protracted anæmia, may result from other causes, in which there is a deficiency in the supply of blood to the nervous centres, spinal cord, or the brain itself. Severe cases of flooding, thoughtless venesection in some cases of epilepsy, the too free use of tart. emet., and many other agents, which may produce nervous derangement and spasm, but to relieve which, we would pursue a directly opposite course from what we would, did the spasm originate from other agents.

In the treatment of anæmia or chlorosis, we may have a vitiated state in the secretions of the stomach, affected spleen, liver, mesentery, or other difficulties, which it may be necessary to correct as far as it is possible; not that they are the seat of the primary affection, but that by correcting these we are able to reach the primary difficulty. Iron, in some of its preparations, has been, and undoubtedly is, the great sheet-anchor in the treatment of these affections; but cases have, and will occur, in which we cannot correct the defects of an anæmic system by iron, however untiringly persisted in, and we are compelled to resort to remedies which will insure a proper restoration of the secretory system, through which we may reach the blood.

CASE 1.—Mrs. C., sixth confinement, aged 34. Patient of an excellent constitution, and capable of much bodily exertion, in easy circumstances, and a good liver. Temperament, nervo-bilious.

Appearance: Body well formed; muscles fully developed; bones large; cheek bones-prominent; eyes bluish-gray; generally of a cheerful disposition. The labor was natural, and, as usual, not very severe or tedious.

In five days able to sit up; by the ninth day assumed the superintendence of the domestic affairs of her household, but not to labor. Fourteenth day took breakfast with the family; at 10 o'clock felt a sudden langour and prostration, which continued for two days; the third day the languor increased; the pulse became one hundred and twenty; eructations from the stomach were frequent, and very offensive; the mouth and tongue were covered with petechial blotches, having ulcerated centres, and leaden-colored bodies; the color deepening towards the base. The tongue was rather dry, with a red streak through the centre, and flattened edges. The urine was voided frequently, with a constant desire during the interval. So extremely offensive were the urine and fæces when voided, that the nurse was compelled to remove them from the room immediately.—The matter voided from the bowels was a dark-green, muco-fæ-

cal, semifluid substance, emitting a fœtor not unlike putrescent dissected flesh. The surface of the body was covered with petechia, not unlike those on the tongue; the sweat, which appeared in paroxysms, was so fetid that it affected the bed and adjoining rooms, to such an extent that it became necessary to use some disinfecting agent, in order to render the patient at all bearable to those present. The eyes were sunken and hollow, yet betraying a wild anxiety about the sudden and fearful calamity which had befallen her. The respiration became oppressive and attended with labored sighings and feelings of syncope, which could only be relieved by vigorous fanning and diffusible stimulants. The position most desired was the left side, but any position was attended with restlessness and constant change. The stomach was irritable, and ejected water and any substance taken into it. There was a difficulty in hearing, with a ringing sound in one ear; the eyes were blurred and vision indistinct. The uterine discharges were stopped, the secretion of milk suspended, and the whole system appeared to be rapidly approaching a state of dissolution. Thus the patient, from a hopeful condition, was in a few hours reduced to one of despair. The succeeding day, the lips, gums, and skin, became pale and waxy; the pulse one hundred and twenty and wiry, with all the phenomena of a distinctly anæmic patient.—Indications. 1st. To relieve the tendency to syncope. 2d, To sustain the system until it could be brought under the influence of medicines.

The syncope, which came on at irregular periods, varying from one to three hours, was immediately relieved by a glass of champagne wine.

The system was sustained by using London porter *ad libitum.* For two weeks, sweetened porter and wine were the only aliments which the stomach would bear.

The alvine discharges were corrected as soon as possible, by repeated doses of Hyd. cum Creta, Pulv. Rad. Rhei, Pulv. Ipe. et Opii. The urinary secretions were corrected by an infusion of Barosma Crenata. The porter acted as a tonic and prophylactic.

The anæmic condition was corrected by the administration of the various preparations of iron. The Ses. Ox., Mur. Tinc., and some of the more concentrated preparations were tried, but the stomach was so sensitive to the impressions of iron, that they were soon ejected, and the treatment necessarily discontinued. Several other tonic remedies were resorted to, but attended with no better success. During this period the stomach would retain the porter and wine, but would in a short time throw off any other food, however prepared.

There was but little tenderness over the bowels; nothing to indicate an active inflammation in any of the viscera. The patient daily became weaker, the pulse more frequent and thready, the countenance cadaverous and anxious, the skin occasionally cold and clammy. Notwithstanding the secretions were more healthy, the anæmic symptoms increased, and everything foreboded a speedy dissolution. At this extremity we resorted to the following preparation:

℞ Argent. Nit. chrys. grs. x.
Ex. Coni. Mac. grs. xx.
Syr. Tolu, oz. ij.
M.

Give a teaspoonful every six hours in rain, or boiled water.

The prescription acted like a charm, in quieting the irritability of the stomach. It gave new life and energy to the system.

The third day after commencing the use of the Nit. Arg. she felt a desire for food, which was relished and retained.

The appetite daily improved, the strength gradually returned, and the patient fully recovered. She has since given birth to a healthy child without any recurrence of anæmia.

CASE 2.—Mrs. J——, æt. 26, confined with her third child, labor healthy and not tedious. Had been troubled with intermitting fever, with some tendency to anæmia, before confinement. For four days after delivery improved rapidly, and everything bid fair for a speedy recovery. At the close of the fourth day she felt greatly exhausted, and a general lassitude throughout her system. When called, I found her much prostrated, with an anxious bleached face. The heart was beating rapidly and laboriously, amounting to severe palpitation of that organ, The bowels were loose, discharging a white, yeasty, fetid matter; the tongue furred and white, indicative of great derangement in the biliary system. Increased anæmia rapidly supervened, and in two or three days the following symptoms were present.

The pulse varied from one hundred and twenty, to one hundred and forty a minute, the lips, gums, tongue, and the whole body, were pale and waxy. The appetite, which before was good, was entirely gone; the thirst was not urgent; the veins could no longer be traced upon the hand, nor filled by pressure.

Tinnitus aurium was especially troublesome, and the bruit de diable was so prominent, that it was audible when sitting near the patient.

The dyspnœa was paroxysmal, and always attended with severe palpitation of the heart. During these paroxysms, which occurred every three or four hours, the doors and windows were necessarily kept open; and so severe did the paroxysms sometimes become, that dissolution appeared inevitable.

The immediate symptoms in these paroxysms were relieved with a glassful of champagne wine, while for a permanent tonic the following was given:

R. Ammoniæ Carb. scrup. j.
Quiniæ Sul. grs. xx.
Camphoræ grs. xv.
Sacch. Alb.
Pulv. Gum Acac. oz. j.
Aquæ oz. iv.
M.

Give a tablespoonful every three hours. In two days the paroxysms were much relieved. The treatment for the anæmia was the same as in the previous case:

R. Argent. Nit. chrys. grs. x.
Ex. Coni. Mac. grs. xx.
Syr. Tolu, oz. ij.
M.

Give a teaspoonful every six hours.

Under this treatment, the patient fully recovered, and has since given birth to a very healthy child, without any return of anæmic symptoms.

I have had repeated opportunities of testing the efficacy of Nit. Argent. in anæmia, and am satisfied it is as much a specific in this disease as are the preparations of cinchona in intermittents.

